# The tyrosine kinase inhibitor imatinib prevents lung injury and death after intravenous LPS in mice

**DOI:** 10.14814/phy2.12589

**Published:** 2015-11-30

**Authors:** R Scott Stephens, Laura Johnston, Laura Servinsky, Bo S Kim, Mahendra Damarla

**Affiliations:** Division of Pulmonary and Critical Care Medicine, Johns Hopkins UniversityBaltimore, Maryland

**Keywords:** Endotoxin, imatinib, lung injury, sepsis

## Abstract

Severe sepsis and septic shock are frequent causes of the acute respiratory distress syndrome, and important sources of human mortality. Lipopolysaccharide (LPS), a component of Gram-negative bacterial cell walls, plays a major role in the pathogenesis of severe sepsis and septic shock. LPS exposure induces the production of harmful reactive oxygen species, and the resultant oxidant injury has been implicated in the pathogenesis of both severe sepsis and ARDS. We previously showed that the tyrosine kinase inhibitor imatinib increases lung endothelial antioxidant enzymes and protects against pulmonary endothelial antioxidant injury. In the present study, we tested the hypothesis that imatinib would protect against lung injury and systemic inflammation caused by intravenous LPS in an intact mouse model of endotoxemia mimicking early sepsis. We found that intravenous LPS induced a significant increase in the activity of lung xanthine oxidoreductase (XOR), an enzyme which is a major source of reactive oxygen species and implicated in the pathogenesis of acute lung injury. Imatinib had no effect of LPS-induced XOR activity. However, pretreatment of mice with imatinib increased lung catalase activity and decreased intravenous LPS-induced lung oxidant injury as measured by *γ*-H2AX, a marker of oxidant-induced DNA damage, lung apoptosis, and pulmonary edema. Imatinib also attenuated systemic cytokine expression after intravenous LPS exposure. Finally, imatinib completely prevented mortality in an in vivo, intravenous LPS mouse model of endotoxemia and lung injury. These results support the testing of imatinib as a novel pharmacologic agent in the treatment of Gram-negative sepsis and sepsis-induced ARDS.

## Introduction

Severe sepsis and septic shock are important causes of human morbidity and mortality (Angus and van der Poll 2013). The incidence of sepsis in the United States is increasing; there are more than 750,000 cases of severe sepsis or septic shock each year with a mortality rate of nearly 30% (Angus et al. 2001; Ani et al. 2015). Sepsis is a major cause of the acute respiratory distress syndrome (ARDS), defined as the acute onset of hypoxemic respiratory failure with bilateral pulmonary infiltrates (Ware and Matthay 2000; Ranieri et al. 2012). Sepsis-associated ARDS portends a particularly poor prognosis (Rubenfeld et al. 2005). Current treatment of severe sepsis, septic shock, and ARDS is supportive. Sepsis therapy consists primarily of intravenous fluid resuscitation, vasoactive agents, and antibiotics (Dellinger et al. 2013). Management of ARDS hinges on low tidal volume ventilation and treatment of the underlying cause (The Acute Respiratory Distress Syndrome Network, 2000; Diaz et al. 2010).

Gram-negative organisms, particularly *Escherichia coli* and *Pseudomonas aeruginosa*, are the most common cause of severe sepsis and septic shock (Vincent et al. 2009; Ani et al. 2015). Lipopolysaccharide (LPS), also known as endotoxin, is a component of Gram-negative bacterial cell walls which plays a major role in the pathogenesis of severe sepsis and septic shock in humans (Cohen 2002). LPS exposure has been shown to induce the production of harmful reactive oxygen species (ROS) (Minamiya et al. 1995; Wiesel et al. 2000; Maitra et al. 2009). Oxidant injury has been implicated in the pathogenesis of both severe sepsis and ARDS (Chabot et al. 1998; Imai et al. 2008; Tang et al. 2008; Galley 2011; Angus and van der Poll 2013; Damarla et al. 2014).

We have previously shown that the tyrosine kinase inhibitor imatinib increases the antioxidant enzyme catalase in pulmonary endothelium and attenuates oxidant injury in both isolated pulmonary endothelial cells and the intact mouse lung (Stephens et al. 2014). Other groups have reported that imatinib attenuates endothelial barrier dysfunction caused by histamine, thrombin, and intratracheal LPS (Aman et al. 2012; Letsiou et al. 2015). We hypothesized that imatinib would protect against lung injury and systemic inflammation caused by intravenous LPS in an intact mouse model of endotoxemia to mimic early sepsis.

## Methods

The Johns Hopkins University Institutional Animal Care and Use Committee approved all animal protocols.

Male C57Bl/6J mice, aged 10–12 weeks were obtained from Jackson Laboratory (Bar Harbor, ME). Mice were maintained by the Division of Comparative Medicine of Johns Hopkins University and were provided food and water ad libitum. All institutional and national guidelines for the care and use of laboratory animals were adhered to.

### Intravenous LPS model

Our murine model of LPS-induced pulmonary vascular injury has been described previously (Damarla et al. 2014). Mice were injected with 5 mg/kg of LPS (0127:B8, product # L3129, Sigma, St. Louis, MO) via the retro-orbital route. This strain of LPS, when given parenterally, has been shown to induce septic shock in C57Bl/6 mice (Xu et al. 1994). For survival studies, mice were weighed daily and followed until death or recovery. Food and water were available ad libitum.

### Imatinib treatment

Imatinib was obtained from Cayman Biochemical (Ann Arbor, MI). Imatinib was dissolved in water and administered to mice via oral gavage at a dose of 200 mg/kg/day (Akashi et al. 2011; Rhee et al. 2011). In the survival experiments, imatinib was administered once daily starting the day before LPS administration (day −1), and continued on day 0, day 1, and day 2. For other endpoints, mice were sacrificed on day 1; imatinib was therefore administered only on day −1 and day 0. Control mice were gavaged with an equivalent volume of water alone.

### Evaluation of lung injury and biochemical studies

For assessment of lung injury and biochemical studies, mice were weighed and sacrificed 24 h after administration of LPS. Blood was collected via cardiac puncture and the lungs were harvested after being flushed with 2 mL PBS into the pulmonary artery and snap frozen in liquid nitrogen. Lung apoptosis was determined by assaying caspase 3/7 activity in whole lung homogenates using the CaspaseGlo assay (Promega, Madison, WI), which measures the activity of caspase 3 and caspase 7 using a proluminescent caspase 3 and 7 substrates. Cleavage of the substrate results in a luminescent signal, which is quantified using a luminometer. The assay was used according to the manufacturer’s directions (Damarla et al. 2014). Lung edema was assessed by determining the ratio of right lung wet weight to total body weight, as described previously (Aggarwal et al. 2010). Xanthine oxidoreductase (XOR) activity was measured in lung homogenates as described previously (Kim et al. 2013a). Catalase activity was measured in lung homogenates using a catalase assay kit (Cayman Biochemical, Ann Arbor, MI) according to the manufacturer’s directions. Western blotting of lung homogenates was performed according to standard techniques, as described previously (Kim et al. 2013a; Stephens et al. 2014). Anti-*γ*-H2AX antibodies were obtained from Cell Signaling (item 2577; Danvers, MA).

### Cytokine measurements

Serum levels of tumor necrosis factor *α* (TNF*α*) and IL-6 were measured using ELISA assays from R&D Systems (Minneapolis, MN) according to the manufacturer’s directions.

### Statistics

Kaplan–Meier survival curves were analyzed using log-rank tests. Other data were analyzed using the appropriate Student’s *t*-test for comparisons between two groups or a randomized one-factor ANOVA with least significant difference post hoc testing to compare mean values from multiple treatment groups. GraphPad 6 software was used for statistical calculations. Values presented in text are means ± SE. Differences were considered significant when *P *<* *0.05.

## Results

### Effect of imatinib on LPS-induced lung XOR and catalase activity

**Figure 1:**
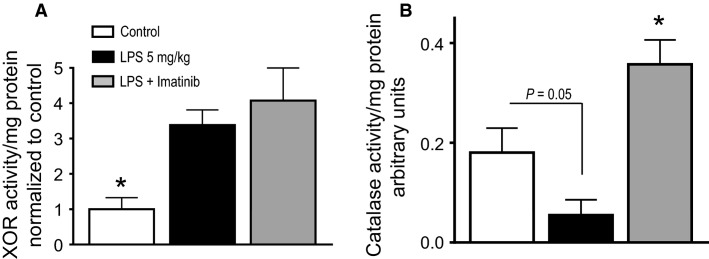
Effect of imatinib on LPS-induced lung XOR and catalase activity. C57BL/6J mice were exposed to IV LPS or PBS (control) for 24 h and lung tissue was harvested for enzymatic assessment. (A) Mice exposed to IV LPS demonstrate a significant (3.38-fold) increase in XOR activity compared to control. A subset of mice pretreated with imatinib and exposed to LPS also shows a significant (4.08-fold) increase in XOR activation. There was no statistical difference in lung XOR activity between mice exposed to LPS alone compared to those pretreated with imatinib and exposed to LPS. (B) Lung catalase activity was reduced in mice exposed to LPS compared to control mice (0.055 ± 0.03 vs. 0.18 ± 0.05). A subset of mice pretreated with imatinib and exposed to LPS showed a marked increase in catalase activity when compared to control mice or those exposed to LPS, 0.36 ± 0.05, 0.18 ± 0.05, and 0.055 ± 0.03, respectively. *N* = 5 per group. One-way ANOVA, *P *=* *0.001. *P <* *0.05 versus all others.

LPS has been reported to induce reactive oxygen species (ROS) generation via the activation of several ROS generating enzymes (Maitra et al. 2009). Xanthine oxidoreductase (XOR), which is an essential enzyme for purine catabolism, is also a major source of ROS. XOR is activated by stressors such as hypoxia, mechanical stress (e.g., high tidal volume ventilation), and inflammatory cytokines; the resultant production of ROS has been implicated in the pathogenesis of acute lung injury (Hassoun et al. 1998; Abdulnour et al. 2006; Boueiz et al. 2008; Kim et al. 2015). To see if intravenous (IV) LPS induced lung XOR activity, we harvested mouse lungs 24 h after IV LPS injection and measured XOR activity. As shown in Figure1A, intravenous (IV) LPS induced a significant threefold increase in lung XOR activity, consistent with prior studies showing that intraperitoneal LPS can induce XOR activity and oxidant lung injury (Hassoun et al. 1998). As also shown in Figure1A, there was no effect of imatinib on LPS-induced XOR activity, indicating that imatinib does not interfere with the LPS-induced activation of XOR and the resultant production of ROS.

Catalase is a key antioxidant enzyme which is essential for protection against lung endothelial oxidant injury (Stephens et al. 2010, 2014). We have previously shown that imatinib increases pulmonary endothelial catalase activity and the ability of pulmonary endothelial cells to scavenge extracellular hydrogen peroxide (H_2_O_2_) (Stephens et al. 2014). Upregulation of catalase protects against oxidant-induced injury in the lung (Christofidou-Solomidou et al. 2003; Kozower et al. 2003; Nowak et al. 2007; Shi et al. 2010; Stephens et al. 2010). We anticipated that lung catalase activity would be increased by imatinib treatment. Catalase activity was measured in lung homogenates 24 h after IV LPS or PBS injection. As shown in Figure1B, 5 mg/kg of IV LPS produced a trend toward decreased lung catalase activity compared to control, though this difference did not reach statistical significance (0.055 vs. 0.18, *P = *0.05). However, in imatinib-treated mice, not only was the LPS-induced decrease in catalase activity prevented, but also catalase activity increased twofold compared to control mice; this increase was significant (0.36 vs. 0.18; *P *<* *0.05).

### Effect of imatinib on LPS-induced lung oxidative damage, apoptosis, and lung edema

**Figure 2:**
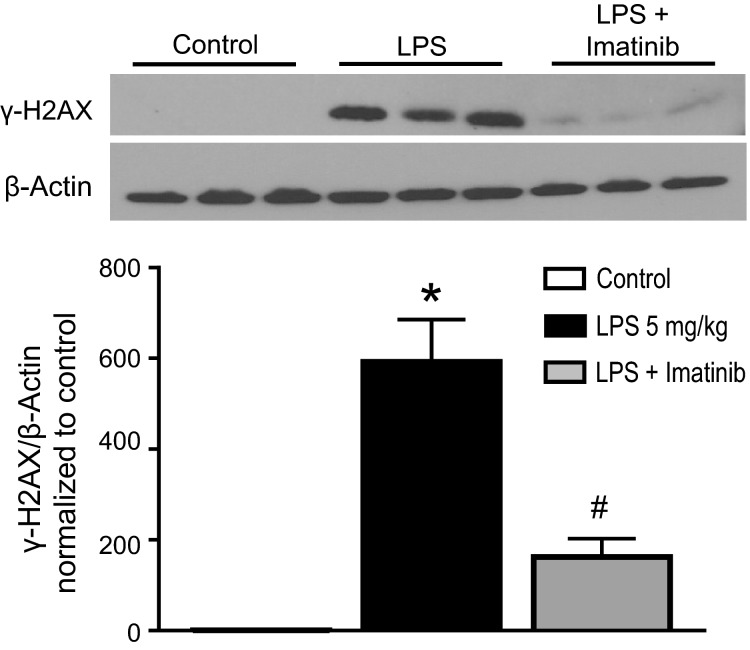
Imatinib prevents LPS-induced pulmonary oxidative damage. C57BL/6J mice were exposed to IV LPS or PBS (control) for 24 h and lung tissue was harvested for markers of oxidative damage. Top panel: Lung tissue homogenates were immunoblotted with antibodies recognizing *γ*-H2AX, a marker of oxidant-induced DNA damage. As shown, there is a marked increase in *γ*-H2AX in mice exposed to LPS. Mice pretreated with imatinib were significantly protected against LPS-induced *γ*-H2AX expression. Bottom panel: Quantification of densitometry confirms a significant increase in *γ*-H2AX expression in response to LPS, which is markedly reduced with imatinib pretreatment. Representative immunoblot of five mice per condition. One-way ANOVA, *P *<* *0.0001. *P <* *0.05 versus control and LPS + Imatinib. ^#^*P *<* *0.05 versus control and LPS.

LPS is known to induce ROS production via activation of several enzymes (Maitra et al. 2009). We show above that LPS induces XOR activity, another source of ROS. In previous in vitro and ex vivo studies, we have previously shown that an increase in catalase levels attenuates oxidant injury in isolated mouse lung endothelial cells and isolated perfused mouse lungs (Stephens et al. 2010, 2014). In order to test whether in vivo catalase activation by imatinib would overcome the pro-oxidant effects of LPS, we assessed for markers of oxidative damage. We used immunoblotting to probe lung homogenates for *γ*-H2AX, a marker of oxidant-induced DNA damage (double-stranded DNA breaks) (Redon et al. 2011; Singh et al. 2015). As shown on the western blot in Figure2, no *γ*-H2AX is present in control lung homogenates, but a robust signal is present after IV LPS exposure. Imatinib nearly wholly eliminates the LPS-induced increase in *γ*-H2AX. Quantities of *β*-actin, used as a loading control, are constant in all samples. Data from densitometry quantification of the western blots, normalized for *β*-actin, are shown in Figure2. IV LPS causes a marked and significant increase in *γ*-H2AX expression; imatinib significantly attenuates this increase, though the level of lung *γ*-H2AX after IV LPS in imatinib-treated mice remains higher than in control mice.

**Figure 3:**
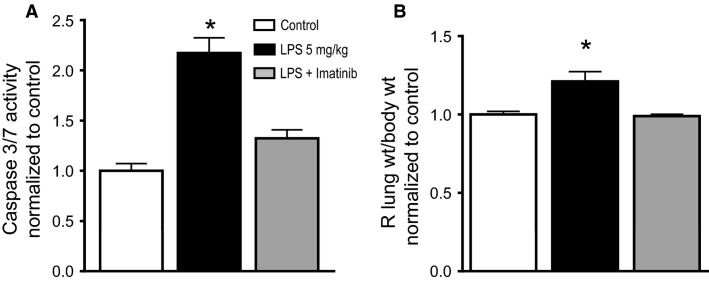
Imatinib prevents LPS-induced lung damage. C57BL/6J mice were exposed to IV LPS or PBS (control) for 24 h and lung tissue was harvested for markers of lung damage. (A) Mice exposed to IV LPS demonstrate a significant (2.2-fold) increase in caspase 3/7 activity compared to control. Mice pretreated with imatinib were significantly protected against LPS-induced caspase 3/7 activation. There was no statistical difference in lung caspase 3/7 activity between control mice and those pretreated with imatinib and exposed to LPS. (B) Mice exposed to IV LPS demonstrated a significant increase in lung edema formation, as assessed by lung wet weight to body weight ratios, compared to control, 4.6 ± 0.23 and 3.8 ± 0.07, respectively. Mice pretreated with imatinib were significantly protected against LPS-induced lung edema formation. There was no statistical difference in pulmonary edema formation between control mice and those pretreated with imatinib and exposed to LPS. *N* = 5–7 per group. One-way ANOVA, *P *<* *0.0001. *P <* *0.05 versus all others.

Oxidant injury to the lungs has several consequences, including increased pulmonary apoptosis and increased vascular permeability (Chabot et al. 1998; Tang et al. 2008). To determine whether imatinib would prevent IV LPS-induced pulmonary apoptosis, we assessed caspase 3/7 activity in lung homogenates 24 h after IV LPS or PBS diluent control. As shown in Figure3A, compared to control mice (injected with PBS), IV LPS significantly increases whole lung caspase 3/7 activity by more than twofold (*P *<* *0.05 vs. control). However, 24 h of pretreatment with imatinib prior to IV LPS completely prevented this increase in caspase 3/7 activity; lung caspase activity after IV LPS in imatinib-treated mice was significantly lower than in mice treated with LPS alone, and not significantly different than control mice (which did not receive LPS). This corresponded to changes in pulmonary edema, as assessed by right lung wet weight/total body weight (Fig.3B). IV LPS caused a significant increase in pulmonary edema, compared to control mice (*P *<* *0.05). This increase in pulmonary edema was completely prevented by imatinib pretreatment.

### Effect of LPS and imatinib on systemic inflammatory cytokine expression

**Figure 4:**
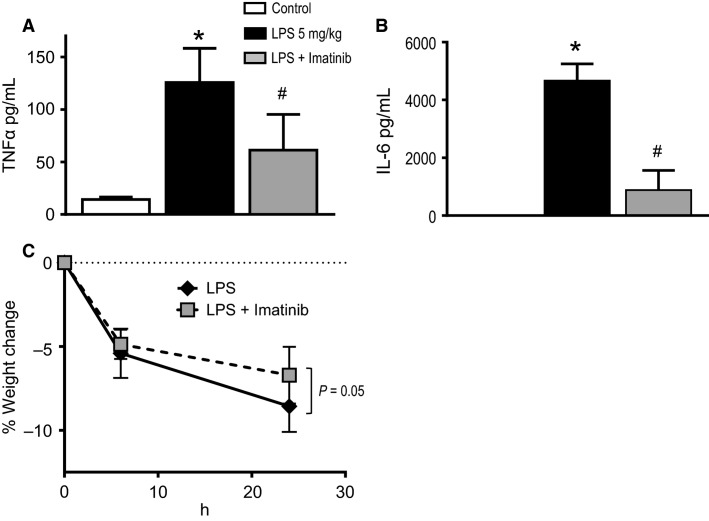
Imatinib prevents LPS-induced systemic inflammation. C57BL/6J mice were exposed to IV LPS or PBS (control) for 24 h and serum was collected for evaluation of cytokines as a marker of systemic inflammation. (A) Mice exposed to IV LPS demonstrate a ∼ninefold increase in serum TNF*α* levels compared to control, 125.6, and 14.25 pg/mL, respectively. Mice pretreated with imatinib were significantly protected against LPS-induced TNF*α* induction. (B) Mice exposed to IV LPS demonstrate a substantial increase in serum IL-6 levels compared to control, 4666 pg/mL, and 0 pg/mL, respectively. Mice pretreated with imatinib were significantly protected against LPS-induced IL-6 induction. (C) Percent weight change from baseline was recorded at 6 h and 24 h after exposure to LPS. As shown, there is a similar amount of weight loss in both LPS-exposed mice and those pretreated with imatinib at 6 h, but by 24 h, mice pretreated with imatinib demonstrate a borderline trend toward reduced weight loss (*P *=* *0.05). *N* = 5–8 per group. One-way ANOVA, *P *<* *0.0001. *P <* *0.05 versus all others. ^#^*P *<* *0.05 versus LPS.

LPS exposure results in the induction of inflammatory cytokines such as TNF*α* and IL-6 (Xing et al. 1993; Le et al. 1998; Lu et al. 2008). In addition, LPS induces the production of harmful ROS (Minamiya et al. 1995; Wiesel et al. 2000; Maitra et al. 2009). Oxidant injury itself can also induce inflammatory cytokines such as TNF*α* and IL-6 (Sharma et al. 2014). These inflammatory cytokines are important contributors to the pathogenesis of severe sepsis, septic shock, and acute lung injury (Frank et al. 2006; Angus and van der Poll 2013). To see whether imatinib would affect cytokine expression in response to IV LPS, we measured levels of the cytokines TNF*α* and IL-6 in serum from control mice, IV LPS-treated mice, and imatinib/IV LPS-treated mice (Fig.4A and B). Minimal TNF*α* and no IL-6 were detected in serum from control mice. IV LPS induced high serum levels of both TNF*α* and IL-6 (125.6 pg/ml and 4666 pg/ml, respectively); these were significantly higher than control mice (*P *<* *0.05). Imatinib pretreatment markedly and significantly attenuated LPS-induced expression of both of these inflammatory cytokines.

To assess the effect of systemic inflammation, mouse weight loss was calculated in response to IV LPS. Mice were weighed at 6 and 24 h after IV LPS. Weight loss data were censored at 1 day after LPS exposure in order to prevent a survival bias which would be the case at later time points. As shown in Figure4C, IV LPS caused equivalent initial weight loss, at 6 h after LPS exposure, in both LPS and LPS plus imatinib-treated mice. However, there was a trend toward divergence of the weight loss curves by day 1 after LPS, with decreased weight loss observed in the imatinib pretreated mice when compared to LPS, though this difference did not reach statistical significance (*P *=* *0.05), at the time of significant differences in systemic inflammatory cytokines Figure4A and B.

### Effect of LPS and imatinib on mouse survival

**Figure 5:**
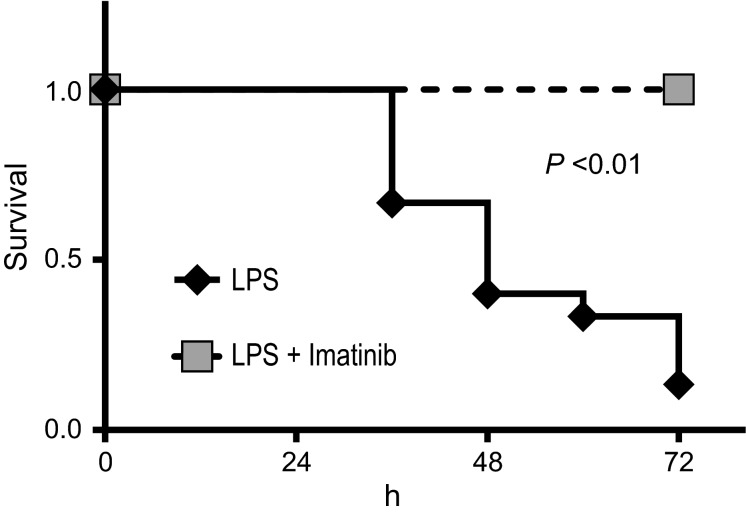
Imatinib prevents LPS-induced mortality. C57BL/6J mice were exposed to IV LPS and followed for mortality. Survival curves for IV LPS and imatinib-treated mice after IV LPS exposure show a significant reduction in mortality with imatinib. *N* = 15 for LPS alone and 5 for imatinib + LPS. Log-rank test with *P *<* *0.05.

Severe sepsis is an important cause of human mortality, killing more than 30% of affected patients (Angus et al. 2001; Ani et al. 2015). To determine whether imatinib would protect against mortality caused by IV LPS, we pretreated mice with either imatinib or diluent (water) via oral gavage. Twenty-four hours later, mice were redosed with imatinib (or water) and injected with IV LPS (5 mg/kg). Imatinib (or water control) was redosed on the subsequent 2 days. Survival curves for IV LPS and imatinib-treated mice after IV LPS are shown in Figure5. All mice survived for the initial 24 h after IV LPS. However, in the control group, mice began dying at 36 h. More than 50% of control mice had died by 48 h after IV LPS, and 90% of mice had died by 72 h after IV LPS. In marked contrast, all imatinib-treated mice survived exposure to IV LPS (*P *<* *0.01 by log-rank test); imatinib provided complete protection against mortality after IV LPS.

## Discussion

Gram-negative sepsis is a common cause of ARDS, and the combination of sepsis and ARDS causes significant mortality. In this study, we report that pretreatment with the tyrosine kinase inhibitor imatinib increases lung antioxidant activity and prevents pulmonary oxidative damage, apoptosis, and lung edema, decreases systemic cytokine expression, and completely prevents mortality after intravenous LPS.

Imatinib was originally developed as an inhibitor of the c-Abl tyrosine kinase, a component of the Bcr-Abl fusion protein which is key to the pathogenesis of chronic myeloid leukemia (CML) (Druker et al. 1996; Braziel et al. 2002; O’Brien et al. 2003). Recently, imatinib has been shown to attenuate pulmonary vascular injury and prevent pulmonary edema in a number of injury models, including thrombin, histamine, vascular endothelial growth factor, oxidant injury, cecal ligation and puncture, and intratracheal LPS (Aman et al. 2012; Kim et al. 2013b; Stephens et al. 2014; Letsiou et al. 2015). To the best our knowledge, however, this is not only the first report demonstrating a protective effect of imatinib in an intravenous LPS model of endotoxemia and lung injury, but also more importantly, this is the first manuscript reporting that imatinib prevents mortality in an animal model of endotoxemia and lung injury.

In our model, imatinib decreased LPS-induced lung apoptosis and pulmonary edema. The decreased lung injury seen in imatinib-treated mice is possibly due to an increase in lung antioxidant capacity, as we show that imatinib has no effect on XOR activity, but rather causes an increase in lung catalase activity and a corresponding decrease in *γ*-H2AX, a marker of oxidant-induced DNA damage. This suggests that the injurious ROS are still generated (at least by XOR), but that the increased levels of lung catalase attenuate the resultant oxidant injury. In work characterizing our IV LPS model of lung injury, we were not able to detect increases in bronchoalveolar lavage (BAL) protein or cell counts after IV LPS. Thus, our results do not appear to be due to an imatinib-mediated alteration of recruitment of immune cells to the lungs after LPS exposure. We cannot definitively say that the protection afforded by imatinib is entirely due to increased antioxidant activity, as it is possible that other protective mechanisms are involved in imatinib-mediated protection against IV LPS. However, our results are consistent with both our prior work, which showed that imatinib both increased levels of catalase and protected against oxidant injury in isolated lung endothelium and intact lungs, and with work by other groups demonstrating that targeted delivery of catalase to pulmonary endothelium can protect against oxidant injury (Christofidou-Solomidou et al. 2003; Stephens et al. 2010, 2014). Specific to LPS-induced lung injury, an increase in catalase decreased lung injury after intranasal LPS exposure (Kumari et al. 2015), and synthetic catalase mimetics attenuated lung injury in endotoxemic animals by preventing ROS-induced injury (Gonzalez et al. 1995, 1996). Interestingly, we found that LPS exposure decreased lung catalase activity compared to control, a finding previously reported only in macrophages (Maitra et al. 2009).

Pulmonary oxidant injury predictably leads to lung endothelial apoptosis, which contributes to pulmonary vascular leak and pulmonary edema (Kawasaki et al. 2000; Lu et al. 2005; Le et al. 2008). Accordingly, we show that imatinib, in addition to increasing lung catalase activity and decreasing *γ*-H2AX expression, decreases lung apoptosis. It is conceivable that this antiapoptotic effect is not wholly due to decreased oxidant injury. LPS can also trigger apoptosis via the Fas/Fas ligand pathway (Perl et al. 2007; Tang et al. 2008), and it is possible that imatinib is interfering with that signaling pathway. However, imatinib actually sensitizes T-cell lymphocytes to Fas ligand-induced apoptotic death (Legros et al. 2012). Barring a cell type-specific effect, this suggests that the antiapoptotic effect of imatinib in our model is not due to an inhibition of an alternative apoptotic trigger, but due to decreased oxidant injury, though we cannot exclude the participation of another antiapoptotic mechanism. It is unlikely that the reduction in LPS-induced pulmonary edema which we observed in imatinib-treated mice is wholly due to a decrease in pulmonary apoptosis, but imatinib has been reported to improve pulmonary vascular barrier dysfunction induced by a variety of injurious stimuli (Rizzo et al. 2015).

We also report that imatinib decreased LPS-induced systemic cytokine expression and attenuated weight loss, a marker of systemic illness. We suspect this is a separate effect of imatinib unrelated to the increased lung catalase activity, as synthetic catalase mimetics prevent ROS-induced lung injury without affecting LPS-induced cytokine expression (Gonzalez et al. 1995, 1996). Imatinib has been reported to inhibit TNF*α* production by monocytes after LPS stimulation (Wolf et al. 2005), and reduced TNF*α* and IL-6 levels after intratracheal LPS in mice (Kim et al. 2013b; Letsiou et al. 2015). IL-6 levels were also reduced in patients with CML who were treated with imatinib (Ciarcia et al. 2012). The mechanism of this cytokine inhibitory effect is incompletely understood, but imatinib is known to inhibit LPS-mediated phosphorylation of the regulatory protein IκB, with subsequent prevention of activation of the transcription factor NF-κB, which is integral to LPS-induced cytokine production (Wolf et al. 2005). We do not know, based on our data, whether the decreased mortality after imatinib treatment is due to decreased lung injury, decreased systemic inflammation (as manifest by decreased cytokine levels), or a combination of the two. Indeed, the two may be inseparable, as inflammatory cytokines are both a marker and cause of lung injury (Frank et al. 2006; Gurkan et al. 2011). A key finding of our study is that IV LPS-induced mortality is decreased by imatinib, a result which has not been reported previously.

Imatinib is approved by the Food and Drug Administration (FDA), and has been in clinical use in humans for more than a decade. Although designed to target the c-Abl tyrosine kinase, imatinib is also a potent inhibitor of several other related tyrosine kinases, including the Abl-related gene (Arg) kinase, platelet-derived growth factor receptor (PDGFR) kinase, and c-kit kinase (Buchdunger et al. 2002). We have previously shown that the effect of imatinib on pulmonary endothelial catalase levels is mediated by c-Abl inhibition (Stephens et al. 2014), and the decrease in inflammatory cytokines in imatinib-treated mice may be due to inhibition of c-Abl, which plays an important role in the activation of macrophages by LPS and subsequent TNF*α* production (Le et al. 1998). However, other groups have implicated the Arg kinase in the barrier-protective effect of imatinib (Aman et al. 2012). It is possible that the antioxidant, barrier-protective, and anticytokine effects of imatinib in our study depend on inhibition of several or all of these related Abl-family kinases.

In conclusion, we found that pretreatment of mice with imatinib (1) increases lung catalase activity; (2) decreases lung oxidant injury, apoptosis, and pulmonary edema; (3) attenuates systemic cytokine expression; and (4) totally prevents mortality in an in vivo, IV LPS model of sepsis and lung injury. These results support the testing of imatinib as a novel pharmacologic agent in the treatment of Gram-negative sepsis and sepsis-induced ARDS.
